# EEG–EMG coupling as a hybrid method for steering detection in car driving settings

**DOI:** 10.1007/s11571-021-09776-w

**Published:** 2022-01-11

**Authors:** Giovanni Vecchiato, Maria Del Vecchio, Jonas Ambeck-Madsen, Luca Ascari, Pietro Avanzini

**Affiliations:** 1 Institute of Neuroscience, National Research Council of Italy, Via Volturno 39/E, 43125 Parma, Italy; 2 Toyota Motor Europe, Brussels, Belgium; 3 Camlin Italy S.R.L., Parma, Italy; 4 Henesis s.r.l., 43123 Parma, Italy

**Keywords:** Hybrid system, Driving, Steering, EEG, EMG, Time–frequency analysis

## Abstract

Understanding mental processes in complex human behavior is a key issue in driving, representing a milestone for developing user-centered assistive driving devices. Here, we propose a hybrid method based on electroencephalographic (EEG) and electromyographic (EMG) signatures to distinguish left and right steering in driving scenarios. Twenty-four participants took part in the experiment consisting of recordings of 128-channel EEG and EMG activity from deltoids and forearm extensors in non-ecological and ecological steering tasks. Specifically, we identified the EEG mu rhythm modulation correlates with motor preparation of self-paced steering actions in the non-ecological task, while the concurrent EMG activity of the left (right) deltoids correlates with right (left) steering. Consequently, we exploited the mu rhythm de-synchronization resulting from the non-ecological task to detect the steering side using cross-correlation analysis with the ecological EMG signals. Results returned significant cross-correlation values showing the coupling between the non-ecological EEG feature and the muscular activity collected in ecological driving conditions. Moreover, such cross-correlation patterns discriminate the steering side earlier relative to the single EMG signal. This hybrid system overcomes the limitation of the EEG signals collected in ecological settings such as low reliability, accuracy, and adaptability, thus adding to the EMG the characteristic predictive power of the cerebral data. These results prove how it is possible to complement different physiological signals to control the level of assistance needed by the driver.

**Supplementary Information:**

The online version contains supplementary material available at 10.1007/s11571-021-09776-w.

## Introduction

Recent advances in sensing and control techniques have made it possible to design cars with a large set of features that can take control of specific aspects of the driving task (e.g., cruise control, autonomous driving) or provide information to the driver to support specific maneuvers (e.g., lane departure). Because driving is a complex behavior involving interrelated motor and cognitive elements such as attention, visuospatial interpretation, visuomotor integration, and decision making (Calhoun et al. 2002; Calhoun and Pearlson 2012), to achieve ever-higher levels of driver support, it is important to investigate and characterize the related brain processes underlying the driver’s actions.

In the years, several attempts were made to characterize the neural correlates of driving in scenarios with different levels of ecology and neural variables, spanning from hemodynamic (Walter et al. 2001; Spiers and Maguire 2007; Mader et al. 2009; Calhoun and Pearlson 2012; Schweizer et al. 2013), to magneto- (Fort et al. 2010; Sakihara et al. 2014) and electrophysiological activity (Schier 2000; Haufe et al. 2011, 2014; Gheorghe et al. 2013; Khaliliardali et al. 2015; Kim et al. 2015; Zhang et al. 2015; Brooks and Kerick 2015; Brooks et al. 2016; Garcia et al. 2017; Vecchiato et al. 2018, 2020). In particular, these latter studies show the possibility of using electroencephalography (EEG) to decode the driver’s cognitive processes in simulated and real car scenarios with the ultimate goal of predicting the upcoming action. Although the success in the classification of salient driving events such as braking (Haufe et al. 2011, 2014; Kim et al. 2014, 2015; Hernández et al. 2018; Wang et al. 2018; Teng et al. 2018; Lin et al. 2018; Nguyen and Chung 2019) and steering (Gheorghe et al. 2013) actions, the level of accuracy is still moderate. Most importantly, the detected neurophysiological features are elicited just around a few milliseconds before the upcoming driving event, making it difficult to implement electronic assisting devices. In fact, despite the noteworthy advancement of recent years, there are still several technological and psychophysiological issues that limit the utilization of the EEG for the real-time identification and monitoring of brain-related activity in driving scenarios. For instance, most EEG sensors remain uncomfortable to place and keep on the head of people who are not used to such recordings, leading to an increase of artifacts and the corruption of the recorded brain signals. Factors such as attention, memory load, and competing cognitive processes (Gonçalves et al. 2006; Käthner et al. 2014; Calhoun and Adali 2016), as well as user’s individual characteristics such as lifestyle, gender, and age (Kasahara et al. 2015) influence brain dynamics producing significant intra- and inter-subject variability (Saha and Baumert 2020; Saha et al. 2021). The low signal-to-noise ratio returned by raw EEG data requires a range of conceptually very different and computationally expensive algorithms to extract significant temporal and frequency EEG features (Müller et al. 2004; Lotte et al. 2007, 2018; Krusienski et al. 2011; Bellotti et al. 2019). Hence, the computing hardware and software must warrant a sufficiently high performance and low latency to preserve the earliness of prediction. For these reasons, it is not always useful to rely on EEG-based predictions alone (Wöhrle et al. 2017). These aspects motivate the need to identify other robust physiological features tackling increased noise due to environmental characteristics and the interaction among neural processes (Lohani et al. 2019). The final aim is to foster the utilization of neurophysiological measurements in experimental settings closer to everyday life activities.

In this sense, surface electromyography (EMG) provides a non-intrusive way of measuring muscle activation and is an appropriate technique when assessing active steering systems (De Luca 1997; Ahlström et al. 2019). It opened new perspectives in ergonomics and provided new tools for analyzing the neuromuscular system in working environments. It is experiencing a growing interest in medical and research applications thanks to the recent availability of novel low-end commercial products increasing the wearability relative to EEG sensors allowing to perform longer recording sessions more comfortably (Gazzoni et al. 2016; Milosevic et al. 2017). Previously, EMG recordings have been used to assess the function of the upper limb muscles during car driving (Jonsson and Jonsson 1975; Liu et al. 2012; Gao et al. 2014). The main findings are that the prime movers are primarily a consequence of steering direction while the stabilizing or fixating muscles are primarily constant, returning that the key muscles correlated to steering are the triceps brachii, the deltoids, pectoralis major, and infraspinatus (Pick and Cole 2006; Liu et al. 2012; Gao et al. 2014). In particular, it is well known that the whole deltoid muscle acts in the abduction of the arm, and there is a synergy between the anterior portion of the muscle and the contralateral posterior portion when moving the steering wheel. More specifically, the anterior portion serves to rotate the steering wheel contralaterally and the posterior portion to rotate it ipsilaterally (Jonsson and Jonsson 1975). EMG can be a reliable method for action prediction because it has limited sensitivity to environmental disturbance (Bi et al. 2019). Compared to EEG, surface EMG can be easily acquired and processed and provide useful information on the movement that the person is executing. Despite the advantages mentioned above, EEG and EMG exhibit different temporal properties concerning a movement onset: changes in the EMG can only be observed close to the actual movement onset, while it is possible to detect EEG motor biomarkers earlier (Bai et al. 2011; Wöhrle et al. 2017; Trigili et al. 2019).

It has been demonstrated that voluntary movements generate changes in the alpha and beta ranges of the EEG spectrum (Pfurtscheller and Lopes da Silva 1999) and that such rhythms are in turn related to modulations of the EMG activity (Hari and Salenius 1999). Recent works have focused on the synchronization between rhythmical activity in the motor cortex and muscular activity employing cortico-muscular coherence, which is usually observed during periods of muscular contraction and has been reported in several studies involving both EEG and MEG (Conway et al. 1995; Boonstra et al. 2009; Cheyne 2013; Rizzo et al. 2020). Current approaches to cortico-muscular coordination focus on associations and synchronous activation between individual brain rhythms at specific cortical areas (e.g., motor cortex, hippocampus), and peripheral muscle activity during specific movement tasks or exercises in ecological conditions (e.g., walking and running) (van Wijk et al. 2012; Cui et al. 2017; Rendeiro and Rhodes 2018; Li et al. 2019a, 2020; Fauvet et al. 2019). In such scenarios, the neuromuscular signals are noisier than those collected in standard laboratory conditions due to uncontrolled environmental settings, and there is also the issue of addressing the simultaneous presence of concurrent cerebral processes. Finally, on the user side, there is the need to create comfortable experimental settings to not pollute the neuromuscular signals with undesired components due to the experienced fatigue of wearing biomedical sensors on the head and several parts of the body. To overcome issues with both EEG- and EMG-based control methods, a combination of both systems, building on each signal’s advantages and diminishing the limitations of each, might be a promising strategy (Lalitharatne et al. 2013). It would then be optimal to identify the cerebral features of interest in standard laboratory settings and then look for neuromuscular invariants in more ecological settings with higher recording complexity. In this way, offline analysis performed on data collected in standard settings would inform the online pipeline to implement to extract neuromuscular features of interest.

Following this reasoning, we propose a hybrid method to distinguish left from right steering in a driving simulator based on EEG and EMG signals collected during steering actions. While using EEG and EMG signals in steering decoding would certainly add predictive power relative to unimodal recordings, such a strategy is hurdled by the complexity of combining the two acquisitions during real-life driving. However, a potential solution could be to first identify in a preliminary recording the steering-related EEG features and then use such information during real driving to increase the predictive power of the steering action based solely on the EMG acquisition.

We used two experimental scenarios to demonstrate this possibility: first, participants were required to perform stand-alone steering wheel movements; successively, we used a driving simulator that represents a step forward towards recording the continuous EEG signals in the real car in natural traffic conditions. This difference between the experimental conditions led us to adopt the terms “non-ecological” and “ecological” steering task. We hypothesized that the electrophysiological correlates of the non-ecological steering could assist the steering action detection in the ecological condition.

Starting from these premises, we first investigated brain and muscular activity underlying steering behavior during the non-ecological steering task. This procedure allowed us to identify the EEG correlates of steering without confounding effects. Then, we correlated such features with the EMG activity collected during a session of driving simulation to extend the validity of the non-ecological cerebral signatures to a more ecological steering task. This double task approach enabled to (i) take advantage of multimodal recordings exploiting the information carried by both neural and muscular data, and (ii) solve ergonomic issues related to the simultaneous acquisition of different signals.

Therefore, we aimed to characterize the EEG–EMG coupling associated with the natural and self-initiated execution of steering actions while driving. To this end, we took advantage of the independent component analysis (ICA), which separates mixed EEG signals into maximally independent activities, each characterized by a precise scalp topography and a corresponding generator pattern, typically modeled as patches of cortical pyramidal cells (Delorme et al. 2012). This strategy also allowed us to avoid field spread caused by the large distance between sensors and neural sources and by the spatial blurring effect of the skull on the scalp’s potential distribution of EEG signals (Schoffelen and Gross 2009). Thus, we expect to identify reliable, independent components whose topography indicates the involvement of motor circuits, whose reactivity precedes the steering action, and is correlated with the muscular activity of the deltoids. Should the EEG–EMG coupling be modulated across the two types of action participants perform (i.e., left and right steering), this would prove that such a hybrid method can be used to discriminate and predict motor intentions associated with natural driving behavior.

## Material and methods

### Participants

Twenty-four participants (6 females, M: 22.8 ± SD: 2.0 years of age) participated in this experiment. They verbally declared that they were right-handed and had a normal or corrected vision, no history of neurologic or psychiatric disorders, and no daily medications. They all have a driving license obtained from the Italian state authority for motor vehicles. None of them had driving experience on professional racetracks. They gave written informed consent to participate in the study consisting of two consecutive sessions comprising a non-ecological steering task and a following ecological steering task. Approval for the study was obtained by the local Ethical Committee (comitato etico Unico per la provincia di Parma).

### Experimental scenarios

#### Non-ecological steering task

**Fig. 1:**
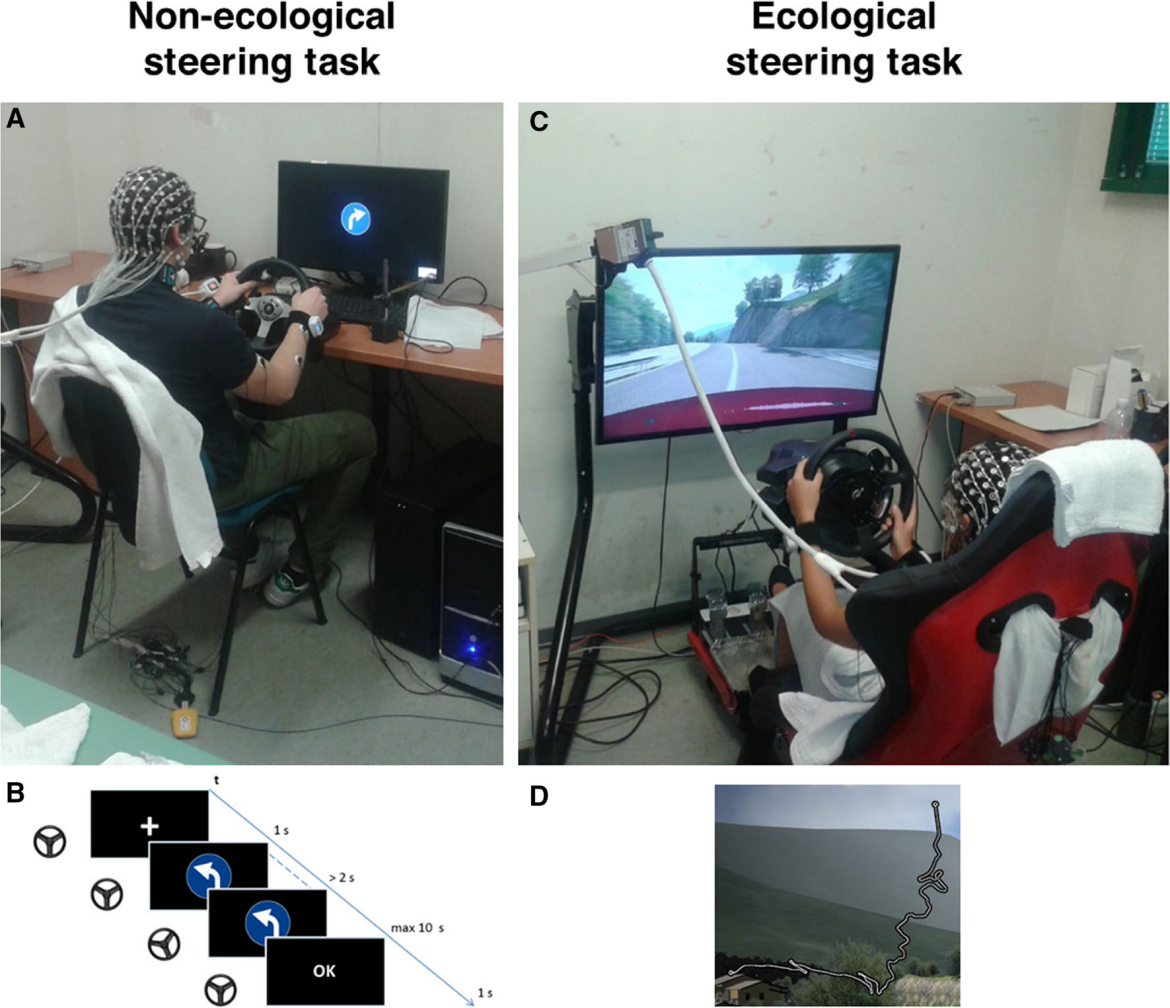
Pictures of the experimental setups related to the non-ecological (panel **A**) and ecological (panel **C**) steering task. Panel **B** shows the timeline of the events during the non-ecological task. Panel **D** presents the road profile of the track used in the ecological steering task

Participants seated in front of a computer screen at a distance of 1 m and were instructed to turn at a quiet pace the steering wheel (Logitech G25) on the right or the left according to a traffic sign randomly presented on display after 1 s from an attentional cue (i.e., a white cross). Participants who performed the steering within 2 s from the traffic sign received an error message and repeated the trial. According to this rule, 88 correct trials have been collected for each participant, equally distributed between left and right steering actions. Synchronization between the visual stimulation, participant’s behavior, and EEG data was realized using timestamps sent by a photodiode placed in front of the screen to capture the task events through white labels onto a black background. Figure 1 shows the experimental setup (panel A) and the time-course of the task (panel B).

#### Ecological steering task

Participants were seated in a driving simulator composed of the RSeat RS1 Assetto Corsa Special Edition (seat) and Thrustmaster T500 RS (steering wheel and pedals). A video game (Assetto Corsa—Kunos Simulazioni) was displayed on a Samsung 40” 5300 class LED TV positioned 1 m from the participant’s seat (vertical field view = 27.4°, horizontal field view = 46.8°). The car used for the experiment was an Alfa Romeo Mito with an automatic transmission. First, participants were asked to get familiar with the environment and the setup by driving for a lap on the Monte Erice track (downloadable at http://assettocorsa.club/mods/tracks/monte-erice.html). Afterward, they performed a single lap on the Coste Loop track (part of the Assetto Corsa software), maintaining the right lane with no particular constraints. They were asked to drive naturally, as they would do in their own car. This circuit simulates a part of the Garda Lake coastal road. No other vehicle was present on both tracks. During the experiment, we collected data related to the steering wheel angle. The synchronization among all the recording devices was implemented with the Lab Streaming Layer (LSL) as described in our previous work (Vecchiato et al. 2018). Figure 1 reports the experimental setup (panel C) and the Coste Loop track (panel D).

### Behavioral data collection and analysis

The steering wheel signals were collected during the non-ecological and ecological tasks and segmented in trials [− 2000, 2000] ms around the steering onset. In order to identify the steering onset in the non-ecological task, we consider the first part of the trial returning wheel angles above the threshold of ± 2°. Of this segment, the time bin corresponding to the point of maximum distance computed from the steering curve and the segment joining the first and last trial sample was identified as the steering onset (see Supplementary Material for a schematic representation of the procedure). When uncertainty in the movement onset arose, we discarded the trial (around 3.5%). This procedure also served for epoching the EEG and EMG signals collected during the non-ecological task into [− 2500, 2500] ms trials around the steering onset.

Instead, in the ecological task, the steering onset was determined by quantizing the steering wheel angle signal. The quantization was realized partitioning the wheel signal with a step of 0.125 between its minimum and maximum. This step was developed to verify that the area-under-the-curve for original and quantized signals differed less than 5%, and their correlation coefficient was above 99%. The quantized signal was binarized setting at 1 all values greater than 0 (in absolute value) and clustered. The steering onset was determined as the time bin corresponding to the transition between zeros and ones. With such procedure, we identified 94.58 ± 31.12 right and 79.08 ± 23.85 left steering trials.

Moreover, the steering wheel signal collected during the ecological task was also segmented during the non-steering intervals in [− 2000, 2000] ms overlapping trials of 1 s, thus identifying 163.71 ± 103.55 trials.

### EEG and EMG data recording and pre-processing

Continuous EEG was recorded in the non-ecological task using the 128-channel Geodesic EEG System (Electrical Geodesics, Inc., Eugene, OR, USA) and the HydroCel Geodesic Sensor Net. Consistent positioning was achieved by aligning the Sensor Net with skull landmarks (nasion, vertex, and pre-auricular points). Using high-input impedance amplifiers (Net Amps300), low-noise EEG data was obtained with sensor-skin impedances maintained below 50 kΩ. The signal was digitized at a sampling rate of 500 Hz (0.01 Hz high-pass filter) and recorded with a vertex reference, the impedance of which was kept below 10 kΩ. Impedances were checked and adapted at the beginning of the ecological steering task. EEG data were exported in raw format using NetStation software (Electrical Geodesics, Inc., Eugene, OR, USA) and then imported into MATLAB to perform the following analysis with EEGLAB v14.1.2 (Delorme and Makeig 2004). The pre-processing comprised line noise removal, bad channels interpolation (1.1 ± 1.1), and common average reference. EEG data were segmented into epochs [− 1.5, 11.5] s around the presentation of the steering traffic sign to consider both the pre-stimulus activity and the one associated with self-paced movements dynamic extending beyond 10 s. Artifacts were rejected by applying a semi-automatic procedure to detect abnormal trends and spectra. On average, we discarded 11.4 ± 7 trials. Clean EEG datasets comprised 37.6 ± 3.7 left and 37.7 ± 3.3 right steering trials.

Continuous EMG data in both non-ecological and ecological steering tasks were acquired using the Neuroelectrics Enobio. EMG signals were sampled at 500 Hz from the left and right deltoids, left and right forearm extensor digitorum as among the main muscles involved in steering actions (Pick and Cole 2006; Lohani et al. 2019) and later imported and processed in MATLAB environment (R2018b, The Mathworks, Natick, MA). EMG data of the non-ecological task were segmented into epochs [− 1.5, 11.5] s around the presentation of the steering traffic sign (as done for the EEG data). In contrast, EMG data of the ecological task were segmented into two datasets: (i) epochs [− 2.5, 2.5] s around the onset of the steering action, as well as (ii) 1 s overlapping epochs of [− 2.5, 2.5] during non-steering intervals. Line noise of the first 5 harmonics of 50 Hz was suppressed using a spectrum estimation technique (Mewett et al. 2001).

To summarize, we have two different segmentations related to the non-ecological ([− 1.5, 11.5] s around the steering sign presentation) and ecological conditions ([− 2.5, 2.5] s around the steering onset). We used different time scales in the results to account for analyses purposes.

EEG data analysis and results related to the ecological steering task were provided as Supplementary Material.

### EMG and EEG data analysis

#### EEG independent component and clustering analysis

We performed an ICA to identify and separate neurophysiological brain activities from other noise sources. On average, we identified 5.9 (± 2.1) independent components (ICs) per subject for a total of 147 EEG ICs. These brain-related IC were identified through equivalent dipole source localization (DIPFIT2) and the utilization of the SASICA EEGLAB plugin (Chaumon et al. 2015). Cluster analysis was performed to group components according to their scalp topographies via the K-means algorithm (Lloyd 1982). The dimension of the cluster space was set to 109, which is the minimum number of good EEG channels remaining across participants after pre-processing. The correlation Squared Euclidean distance is the metric used for minimization measure was chosen to compute cluster centroids using the following formula:

$${\text{d}}\left( {{\text{x}},\,{\text{c}}} \right)\, = \,\left( {{\text{x}}\, - \,{\text{c}}} \right)\left( {{\text{x}}\, - \,{\text{c}}} \right)^{\prime } ,$$

where x is the vector identifying the single-subject scalp map and c is a centroid. The number of clusters was chosen by a bootstrap-based method, leading to the computation of a stability index (Ben-Hur et al. 2002; Salvador and Chan 2004) and identifying 5 clusters. The rationale of using this data-driven analysis was to identify those brain components related to steering actions. Hence, the cluster analysis allowed grouping those EEG ICs related to different cerebral processes, while the statistical comparisons between left and right conditions allowed identifying the cluster reacting to steering actions. Details related to the pre-processing pipeline, ICA, and clustering can be found in our previous work (Vecchiato et al. 2018).

#### Time–frequency analysis

For each EMG signal and EEG IC within the identified clusters, we computed the event-related spectral perturbation (ERSP) as time–frequency decomposition using Morlet wavelets (Makeig et al. 2004). We used frequencies that increased from 2 to 200 Hz in 198 linearly spaced steps for the EMG data, with the number of wavelet cycles increasing from 3 to 60 in linear steps. We used frequencies that increased from 2 to 40 Hz in 38 linearly spaced steps for the EEG data, with the number of wavelet cycles increasing from 3 to 12 in linear steps. Then, dB conversion was performed with a single-trial baseline normalization using the [− 0.5, − 0.1] s time window before the steering sign presentation for the non-ecological steering task and the interval of [− 2, 2] s for the ecological paradigm.

This procedure allowed us to investigate EEG and EMG correlates of steering actions in both time and frequency domains.

#### Cross-correlation analysis

To identify the possible time lag between the EEG signals recorded in the non-ecological task and the EMG signal recorded in the ecological task, we computed the bi-dimensional cross-correlation between the corresponding time–frequency panels using the *xcorr2* function provided by Matlab, which is based on a bi-dimensional convolution between the two input matrices. In particular, for each subject and steering condition in the non-ecological task, we extracted the EEG mask identified within the time–frequency panel around [8, 20] Hz and [− 1.5, − 1] s before the steering onset (e.g., left non-ecological steering), whose dimension is 13 × 50 (frequency x time). Then, we performed the bi-dimensional cross-correlation trial-by-trial for each subject between such EEG mask and the EMG time–frequency panel within the time range [− 1, 2] s (e.g., left ecological steering), whose dimension is 198 × 300. The output of this calculation is a matrix of dimension 210 × 349, from which we discarded the first 12 rows related to non-significant frequencies. Hence, the final cross-correlation matrix used for the following statistical analysis had dimension 198 × 349.

The specificity of the resulting cross-correlation patterns for the steering actions was addressed by computing the same analysis using the EMG trials related to non-steering intervals as defined in the previous section. In particular, EMG non-steering trials were randomly assigned to left and right pseudo-steering conditions with a half split technique: for each of 300 iterations, non-steering trials were divided into two sets, only one of these subsets was used to create the left and right pseudo-steering conditions. Then, for each iteration, we computed the cross-correlation values with the EEG mask resulting from the analysis of the non-ecological dataset. Finally, we also tested the specificity of the cross-correlation patterns for the directionality of the steering by adopting a shuffling procedure. For each of 300 iterations, we created the pseudo-left and pseudo-right conditions by randomly assigning EMG trials from the original left and right steering datasets to the two pseudo-steering conditions and then computing the cross-correlation values.

This procedure allowed us to estimate the contribution of the EEG mask in predicting the EMG activity of the single muscles (i.e., deltoids and forearms extensors) and experimental conditions (i.e., left vs. right steering, steering vs. non-steering, steering vs. shuffled-steering).

#### Statistical analysis

To discriminate the EEG and EMG activity in both non-ecological and ecological tasks, the corresponding time–frequency panels were compared using dependent sample t-statistics and non-parametric permutation testing, corrected for multiple comparisons by weighted cluster mass correction with randomization of 1000 and a statistical threshold of 0.05 (Hayasaka and Nichols 2004; Maris and Oostenveld 2007). The statistical comparison between left and right EEG IC ERPSs in the non-ecological task allows identifying and extracting the time–frequency EEG features associated with steering actions and is used for the following cross-correlation analysis. The significance of the t-statistics computed to test the specificity for steering and directionality was assessed by comparing the observed statistics to the statistical properties of the null-hypothesis distribution. Hence, the observed test statistic values were converted into Z scores, and then the corresponding p-values were computed and reported (Cohen 2014).

This procedure allowed us to assess the significance of the predictive power of the EEG mask for steering actions and directions.

## Results

### Non-ecological steering task

**Fig. 2:**
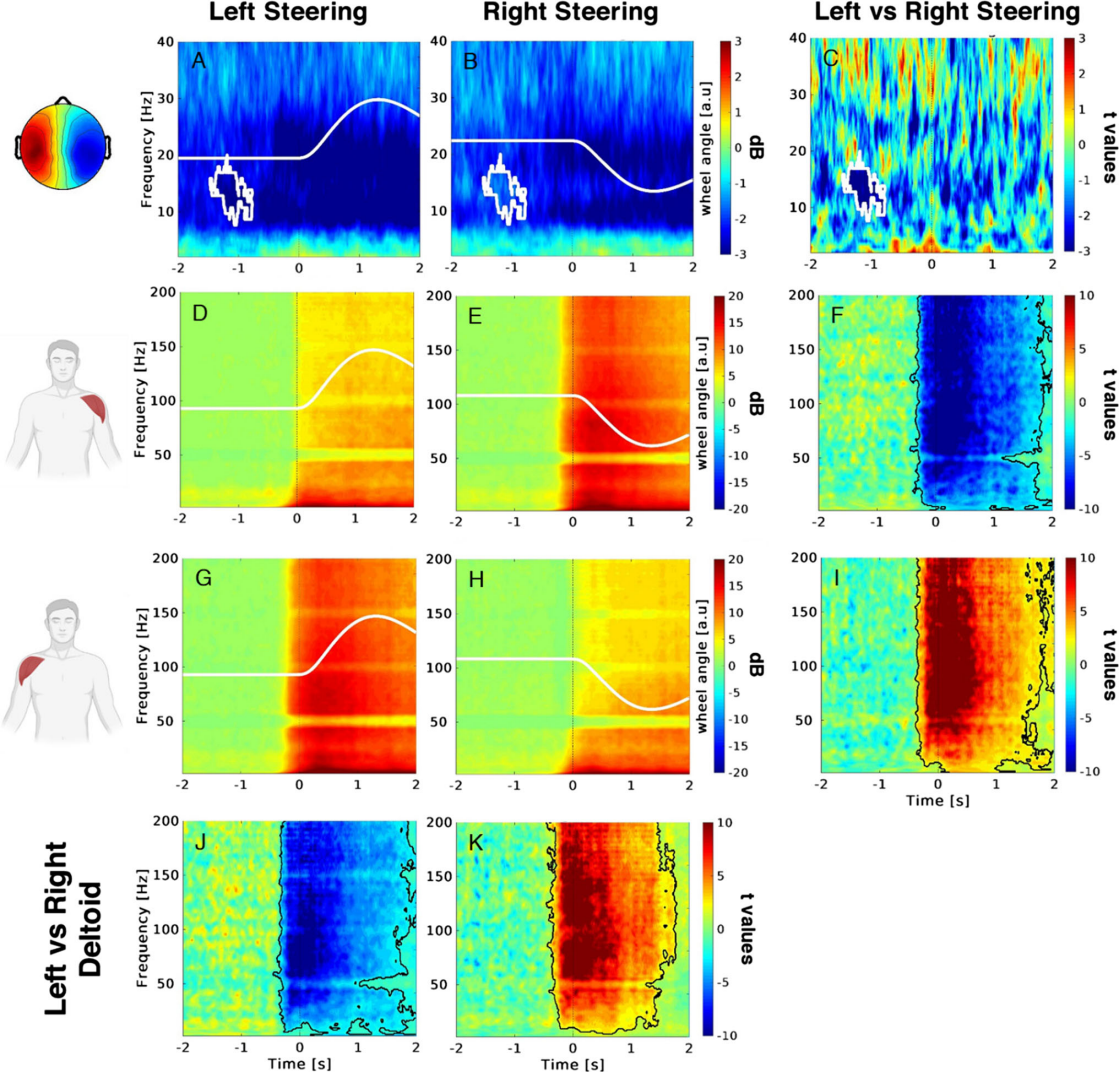
ERSP for the EEG IC and EMG signals collected during the non-ecological steering task. The first three rows (from the top) illustrate the ERSP for the left (**A**, **D**, **G**), right (**B**, **E**, **H**), and the statistical comparison of the two conditions (**C**, **F**, **I**) for the EEG IC, EMG of the left and right deltoid, respectively. The topography in the left part of the picture shows the average scalp map related to the cluster 3 centroid reacting to steering actions. The lower row illustrates the statistical comparisons of the EMG ERSP between left and right deltoid during left (**J**) and right (**K**) steering. Colorbars indicate EEG IC and EMG activity variations relative to the baseline and the corresponding t-statistics. White lines depict the left and right steering wheel angle profiles (**A**, **B**, **D**, **E**, **G**, **H**). White mask (**A**–**C**) delimits the statistically significant portion of the EEG IC ERSP panel. Black mask (**F**, **I**, **J**, **K**) delimits the statistically significant portion of the EMG ERSP of the corresponding compared panels (non-parametric t-test, cluster corrected)

For each cluster of EEG ICs, we performed the non-parametric permutation test to compare the ERSP between the two steering conditions (i.e., left vs. right) in the time window of [− 2, 2] s relative to the onset of the event. The analysis revealed a statistically significant difference onset only for cluster 3, highlighting a de-synchronization of the mu rhythm around 1.5 s before the left steering. This cluster is populated by 24 ICs belonging to 16 subjects (illustrated in the Supplementary Material). Figure 2 shows the scalp topography of such a significant cluster along with the modulation of the related time–frequency activity during left (panel A) and right (panel B) non-ecological steering with the corresponding t-statistics (panel C). Centroid maps with the corresponding time–frequency panels of the non-significant EEG IC clusters are illustrated in the Supplementary Material.

Figure 2 also shows the ERSP of the EMG activity for the left (panels D, E) and right deltoids (panels G, H) during the left (panels D, G) and right (panels E, H) steering. Specifically, when comparing these signals between left and right steering, we observed the significant broadband increase of the muscular activation of the left deltoid during the right steering (panel F), as well as the symmetrical broadband increase of activity of the right deltoid during the left steering (panel I). Analogously, when comparing these signals between left and right deltoids, we observed the significant broadband increase of the muscular activation of the right deltoid during the left steering (panel J), as well as the symmetrical broadband increase of activity of the left deltoid during the right steering (panel K). The statistical comparisons of the muscular activation of the two forearm extensors did not return significant differences in the EMG broadband as the two deltoids (see Supplementary Material, Figure S3); thus they are not considered in the following analysis.

Variations of the wheel angle for the left and right steering were represented with the white signals within each corresponding panel of Fig. 2.

### Ecological steering task

**Fig. 3:**
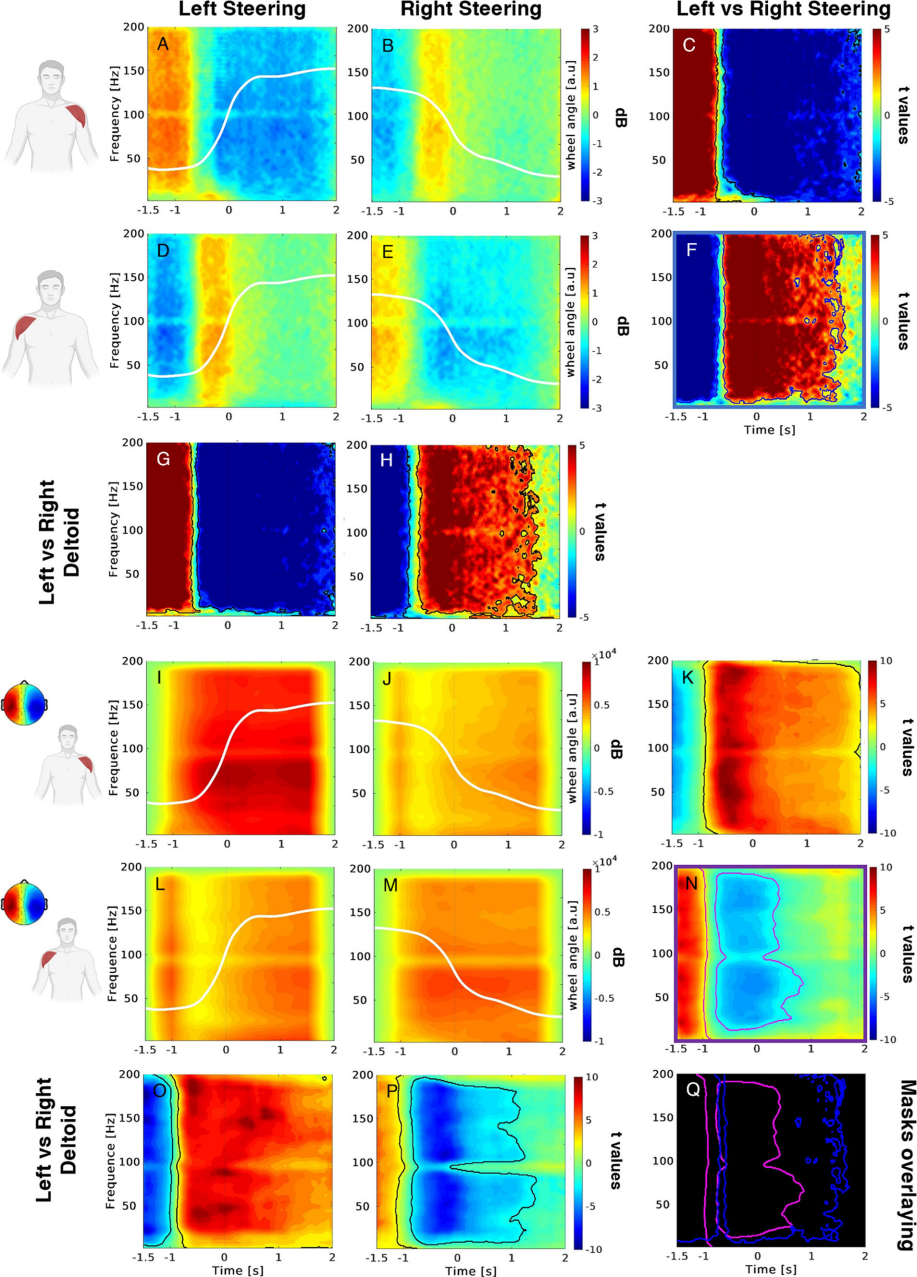
ERSP for the EMG signals collected from the deltoids during the ecological steering task (upper box), and cross-correlation results between EEG IC and EMG data (lower box). Upper box. The first and second rows (from the top) illustrate the EMG ERSP for the left and right deltoid during left (**A**, **D**) and right (**B**, **E**) steering and the statistical comparison of the two conditions (**C**, **F**). The third row illustrates the statistical comparisons of the EMG ERSP between left and right deltoid during left (**G**) and right (**H**) steering—lower box. The first and second rows (from the top) illustrate the EEG–EMG cross-correlation values for the left and right deltoid during left (**I**, **L**) and right (**J**, **M**) steering and the statistical comparison of the two conditions (**K**, **N**). The third row illustrated the statistical comparisons of the EEG–EMG cross-correlation values between left and right deltoid during left (**O**) and right (**P**) steering. The topography in the left part of the picture shows the average scalp map related to the cluster 3 centroid reacting to steering actions and used for the EEG–EMG cross-correlation. Color bars indicate variations of EMG activity (upper box) and the EEG–EMG cross-correlation (lower box) relative to the baseline and the corresponding t-statistics. White lines depict the left and right steering wheel angle profiles (**A**, **B**, **D**, **E**, **I**, **J**, **L**, **M**). Contour mask (**C**, **F**, **G**, **H**, **K**, **N**, **O**, **P**) delimits the statistically significant portion of the corresponding compared panels (non-parametric t-test, cluster corrected). Panel **Q** highlights the significant masks corresponding to the comparisons left versus right steering for the right deltoid for the EMG ERSP data (blue contours, **F**) and EEG–EMG cross-correlation (magenta contours, **N**). (Color figure online)

Figure 3 shows the results related to the analysis of the EMG signals during the ecological task performed at the driving simulator. In particular, the upper box presents the ERSP computed for the EMG signals of the left and right deltoids during left and right steering actions. The different panels of this figure are arranged as Fig. 2 to show that the asymmetrical activation of the two deltoids during steering observed in the non-ecological task is replicated in the ecological condition. Specifically, we highlight the significant broadband increases of the muscular activation of the left deltoid during the right steering (panels A–C) and the symmetrical broadband increase of activity of the right deltoid during the left steering (panels D–F). Analogously, when comparing these signals between left and right deltoids, we observed the significant broadband increase of the muscular activation of the right deltoid during the left steering (panel G), as well as the symmetrical broadband increase of activity of the left deltoid during the right steering (panel H).

The lower box of Fig. 3 shows the results related to the cross-correlation analysis performed between the EEG mask gathered during the non-ecological task and the EMG ERSP panel corresponding to the ecological scenario in the same steering condition and for each of the two deltoids (i.e., non-eco EEG in left steering cross-correlated with the eco EMG in left steering). Here we can observe higher cross-correlation values associated with the left deltoid during left steering (panel I) and for the right deltoid during the right steering (panel M) when compared with the right (panel J) and left (panel L) steering, respectively. This pattern is statistically demonstrated with the corresponding non-parametric analysis (panel K, N). Moreover, such differences between cross-correlation values are also observed during left (panel O) and right (panel P) steering when comparing the left and right deltoid. Strikingly, these statistics show that significant activations related to the cross-correlation analysis are detected earlier (magenta contour in panel Q) relative to the activations of only EMG ERSP (blue contour in panel Q), for the condition right deltoid, left versus right steering.

**Fig. 4:**
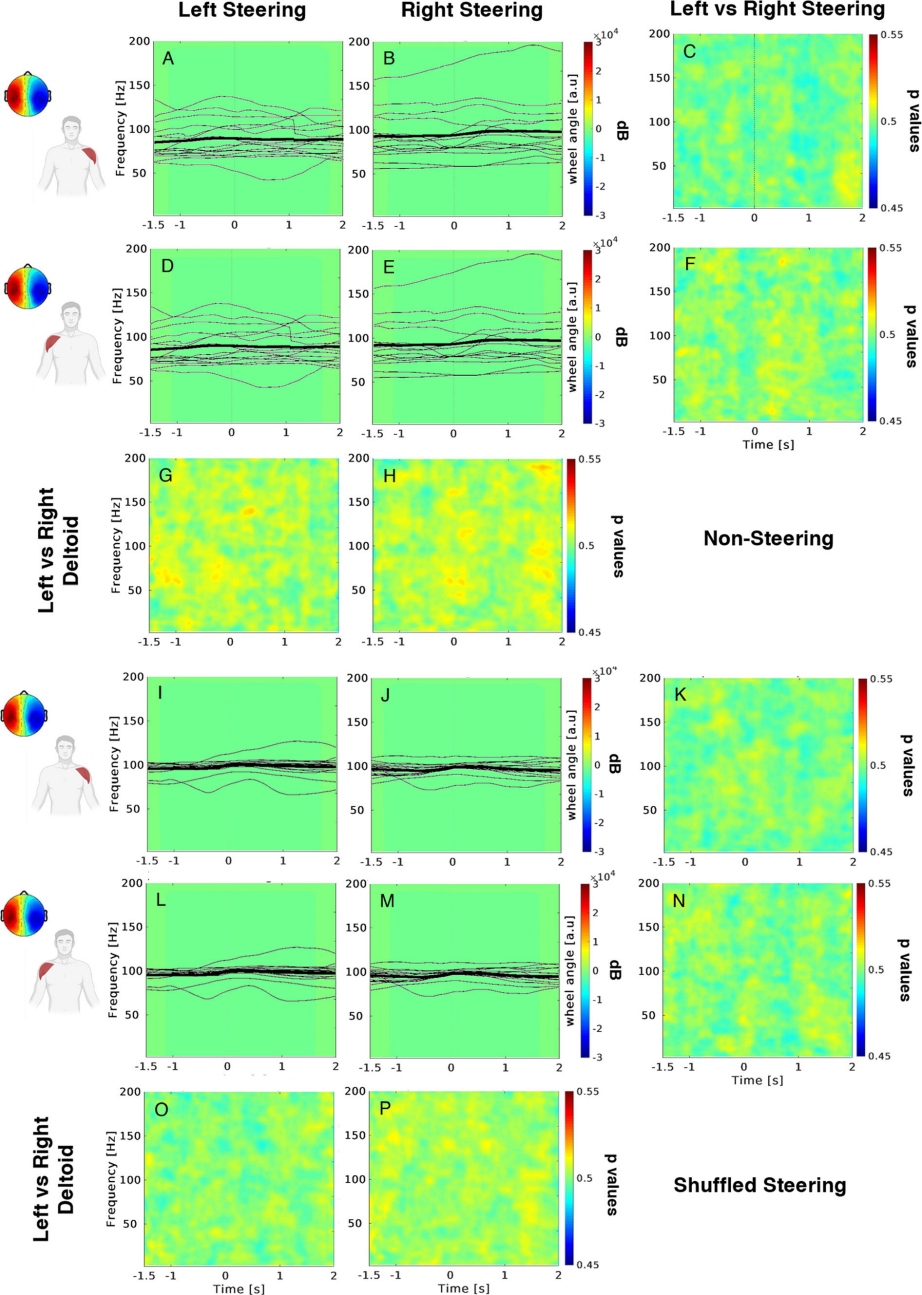
Cross-correlation results between EEG IC and EMG data related to non-steering (upper box) and shuffled steering (lower box) conditions. Same convention as Fig. 3 (lower box). Black lines depict the butterfly plot of left and right steering wheel angle profiles. Thick lines represent the average across subjects (thin lines)

Figure 4 shows the results of the cross-correlation analysis performed between the same EEG mask gathered during the non-ecological task, and the EMG ERSP panels corresponding to the ecological scenario in the non-steering (upper box) and shuffled-steering (lower box) conditions. Here we can observe low cross-correlation values associated with all conditions reported on the same scale of Fig. 3 (lower box). Finally, the corresponding statistics returned no significant difference for any condition.

Variations of the wheel angle for the left and right steering were represented with the white (Fig. 3) and black (Fig. 4) signals within each corresponding panel.

The analysis of the EEG collected during the ecological steering task did not return any significant cluster related to cortical components reacting to steering actions. Thus, we report the corresponding ERPSs and statistical comparisons in the Supplementary Material.

## Discussion

In the present study, we report that the modulation of the EEG mu rhythm observed during the motor preparation of non-ecological steering predicts the muscular activity of deltoids, thus anticipating subject steering behavior. The reactivity of such rhythm measured across sensorimotor areas during the non-ecological steering preparation anticipates the corresponding action. This result paves the way for using such a cerebral feature to discriminate steering actions in the ecological task. We report the increase of EMG activity of the deltoid anticipating the contralateral steering in non-ecological and ecological steering tasks. These results show an asymmetric muscular activity of the deltoids beginning before the action onset remaining steady during the steering execution, i.e., the coordinated increase of power of the right deltoid and the corresponding decrease of power of the left deltoid is associated with the steering action on the left side. Such findings show that monitoring the two deltoids’ muscular activity makes it possible to discriminate the steering side before the action onset while driving in non-ecological and ecological scenarios. Strikingly, the identified non-ecological EEG feature correlates with the ecological EMG activity of the deltoids, providing an improvement of the discrimination power of the steering side during driving simulation. The comparison between the results related to the EMG analysis and those concerning the EEG–EMG cross-correlation returned larger relative timing in favor of the latter related to the succession of steering events. In fact, on one side, there is the issue of distinguishing the anticipatory components due to EEG predicting power from those which are merely due to computational accounts, e.g., windowing implied in the cross-correlation calculation. However, we observe a clear anticipatory pattern returned by cross-correlation values related to the succession between left and right steering events. This change of cross-correlation values is detectable in advance relative to the single EMG signal.

The timing of the EEG response identified in this study is compatible with patterns of event-related de-synchronization (ERD) which are reported to be predictive of the upcoming action between 1.5 and 1 s before the movement onset (Neuper et al. 2006). ERD reflects non-phase-locked EEG changes of oscillatory activity within the alpha and beta frequency due to sensorimotor events (Pfurtscheller and Lopes da Silva 1999; Savić et al. 2020). The EEG component identified during the non-ecological task arises from the motor areas and may reflect the preparation of steering actions. The scalp topography that we reported is reminiscent of the mu rhythm observed in motor and premotor regions (Gastaut et al. 1952; Pfurtscheller et al. 1997). Such electrical activity produces somatotopically organized de-synchronization during execution, observation, and imagination of actions (Pineda 2005; Pfurtscheller et al. 2006; Arnstein et al. 2011; Avanzini et al. 2012). These regions perform several functions other than body movements control, such as sensory-motor transformation, action understanding, decision-making regarding execution and initiation of action, preparation, and planning of complex movements (Roland 1984; Rizzolatti and Luppino 2001). Recent literature reports ERD in contralateral sensorimotor cortices during movement preparation of visually cued movements (Li et al. 2018; Little et al. 2019). Therefore, we argue that the identified EEG mu rhythm modulations regulate the motor preparation of the right and left deltoids for steering actions. In particular, we report that the mu de-synchronization over left sensorimotor regions is related to the right deltoid’s activity during left steering. This would show that left steering involves larger neural computation than right steering, despite the absence of significant difference in the steering wheel angle, as already reported in a previous study (Oka et al. 2015). Another study also reports the de-synchronization of the alpha rhythm across sensorimotor regions related to relative steering angle compensation (Brooks and Kerick 2015), thus showing the relation between such electroencephalographic feature and steering response as already observed in more simple tasks (Pfurtscheller and Neuper 1994; Stancák and Pfurtscheller 1996).

Analyzing the EMG in the time–frequency domain, we observe that the maximum muscle activity changes significantly due to different steering wheel angles and turning directions in non-ecological and ecological scenarios. It is known that the anterior and middle portions of the deltoid muscle work intermittently during car driving and that their functioning regulates the contralateral rotation of the steering wheel, being activated for a duration of around 50% since the initial stage of the action (Jonsson and Jonsson 1975; Pick and Cole 2006; Liu et al. 2012; Gao et al. 2014). Specifically, muscle activity is relatively small when the steering wheel is near its center, but it increases rapidly as the wheel starts to turn (Gao et al. 2014). Although EMG was successfully used to identify the muscles involved in generating and predicting torque at the steering wheel (Pick and Cole 2006), EMG alone has low power in predicting the steering as we report to be limited to a few hundred milliseconds. Thus, we exploited the EEG scalp feature to improve steering detection by computing the cross-correlation between the mu rhythm de-synchronization retrieved during the non-ecological task and the EMG activity elicited in the ecological scenario. This procedure allowed us to enlarge the time window in which it is possible to discriminate the steering action. We showed that this EEG–EMG coupling is specific for steering actions and their directionality, returning better results relative to single EMG signals. Similar findings were recently reported concerning the use of a hybrid human–machine interface for gait decoding (Tortora et al. 2020), motor rehabilitation of stroke patients (Sarasola-Sanz et al. 2017), movement detection of hand-paralyzed patients (Lóopez-Larraz et al. 2018). All these studies suggested an increase in the classification accuracy and the number of commands for human–machine-interfaces (Hong and Khan 2017).

Here we performed two consecutive recording sessions to demonstrate the feasibility of exploiting EEG correlations of steering behaviour collected in a standard highly controlled environment to detect steering action in a more ecological setting such as a driving simulator. In the first non-ecological recording session, we extracted ERD as a neural correlate of the EMG activity of the deltoids predicting the upcoming steering behavior. In the following ecological recording session performed at the driving simulator, we collected the EMG activity of the deltoids. We showed that the ERD resulting from the previous session is indeed informative concerning the steering action in this more naturalistic scenario. As expected, a large variability characterized the EEG collected during the ecological steering task, and the statistical analysis did not return any significant result related to the cerebral steering component. Indeed, steering actions were reliably discriminated through the analysis of the cortical correlates associated with the non-ecological task. This evidence suggests the usefulness of exploiting cortical correlates of motor preparation of non-ecological actions to predict the same action in ecological conditions. The non-significant results of the EEG collected during the ecological steering task indeed highlight the usefulness of exploiting cortical correlates associated with the non-ecological task for steering detection in a more natural condition. From a methodological perspective, we used the ERSP to investigate EMG correlates of steering for directly assessing the coupling of cerebral and muscular activations. EMG features in the time domain, such as Root Mean Square and Mean Absolute Value, could also be useful for future analyses implementing online classifiers.

Several recent studies on driving addressed the issue of steering classification using different EEG features. In particular, it was assessed the possibility to decode self-generated actions detecting whether the driver would perform a lane change shortly in a simulated highway (Gheorghe et al. 2013). Authors report slow negative EEG deflections across central areas consistent with the movement-related potentials 500 ms before the lane change, yielding a classification accuracy of 79% with an average detection time of 613 ms before the actual steering action. In another series of studies performed with both driving simulators and real cars (Zhang et al. 2015), error-related brain potentials were analyzed to investigate the possibility of using an external device to be adapted to the driver’s goal, i.e., assisting in the upcoming steering action. This strategy was enacted by showing the drivers a visual stimulus indicating their inference about the next turning direction when approaching an intersection. Authors report differences in the EEG response over fronto-central areas when the directional stimulus does not match the driver’s intention. Statistical differences between error and correct conditions were observed between 200 and 600 ms after feedback, yielding a mean accuracy of the event-related decoding of 0.68, which indicates the possibility of extracting meaningful information about the driver’s need for assistance.

Several studies demonstrated that the combination of EEG and EMG can improve the reliability of movement prediction based on a single modality (Vecchiato 2021; Di Liberto et al. 2021). For example, the prediction of movement onset based on EEG analysis can be improved by designing hybrid systems monitoring at the same time additional peripheral signals depending on the context requirements. EEG and EMG signals can reliably predict movements before the action onset, showing that both can potentially control an electronic device (Kirchner et al. 2014). Different measures could be combined for different movement stages, e.g., movement planning, the start of the movement, and movement execution (Novak et al. 2013). Other investigations were conducted to predict voluntary movements before their occurrence (Bai et al. 2011) and vehicle steering (Gomez-Gil et al. 2011) using hybrid EEG–EMG BCIs. The hybrid strategy was initially introduced in the Brain Computer Interfaces (BCIs) to exploit the advantages of different physiological signals and computational approaches to achieve specific goals better than a conventional EEG-based system (Pfurtscheller et al. 2010; Li et al. 2019b). A hybrid system might predict user intention with higher accuracy, thus improving the whole system’s performance or reducing the rate of false positives (Usakli et al. 2009, 2010; Ma et al. 2015; Hong and Khan 2017). Today such a methodology is used to boost motor rehabilitation of stroke patients (Sarasola-Sanz et al. 2017), for the online movement prediction (Kirchner et al. 2014; Wöhrle et al. 2017), gait decoding (Tortora et al. 2020), and its application is investigated in other several domains of bio-robotics (Lalitharatne et al. 2013).

The existence of preparatory electrophysiological activity elicited before the onset of steering action allows us to infer the upcoming driving actions in advance. The reported EEG–EMG coupling is a proof of concept for utilizing hybrid systems for the detection and online prediction of driving actions, exemplifying how it might be possible to complement information from behavioral, physiological, and external sources to control the level of assistance needed by the driver in that context (Chavarriaga et al. 2018). The predictive power of the EEG–EMG coupling demonstrated in a car simulator could be further investigated in larger sets of actions to extend the validity of this neurophysiological mechanism beyond driving.

## Supplementary Information

Below is the link to the electronic supplementary material.

Supplementary file1 (DOCX 9065 kb)

## Data Availability

The data that support the findings of this study are available from CAMLIN Limited but restrictions apply to the availability of these data, which were used under licence for the current study, and so are not publicly available. Data are however available from the authors upon reasonable request and with permission of CAMLIN Limited.

## Code availability

Custom code is available upon reasonable request.
