# Supplementary material for: EEG–EMG coupling as a hybrid method for steering detection in car driving settings
Source: Cogn Neurodyn. 2022 Jan 11;16(5):987–1002. doi: 10.1007/s11571-021-09776-w (PMC9508316; doi:10.1007/s11571-021-09776-w)
Supplement: Supplementary file 1 — Supplementary file1 (DOCX 9065 kb) [file 11571_2021_9776_MOESM1_ESM.docx]

**Supplementary Material**

**EEG-EMG coupling as a hybrid method for steering detection in car driving settings**

Giovanni Vecchiato, Institute of Neuroscience, National Research Council of Italy, Parma

https://orcid.org/0000-0001-6037-5806

Maria Del Vecchio, Institute of Neuroscience, National Research Council, Italy, Parma

Jonas Ambeck-Madsen, Toyota Motor Europe

Luca Ascari, Camlin Italy s.r.l.

Pietro Avanzini, Institute of Neuroscience, National Research Council, Italy, Parma

Corresponding author: Giovanni Vecchiato, Institute of Neuroscience, National Research Council of Italy, Via Volturno 39/E, 43125, Parma, Italy. [giovanni.vecchiato@in.cnr.it](mailto:giovanni.vecchiato@in.cnr.it)

**
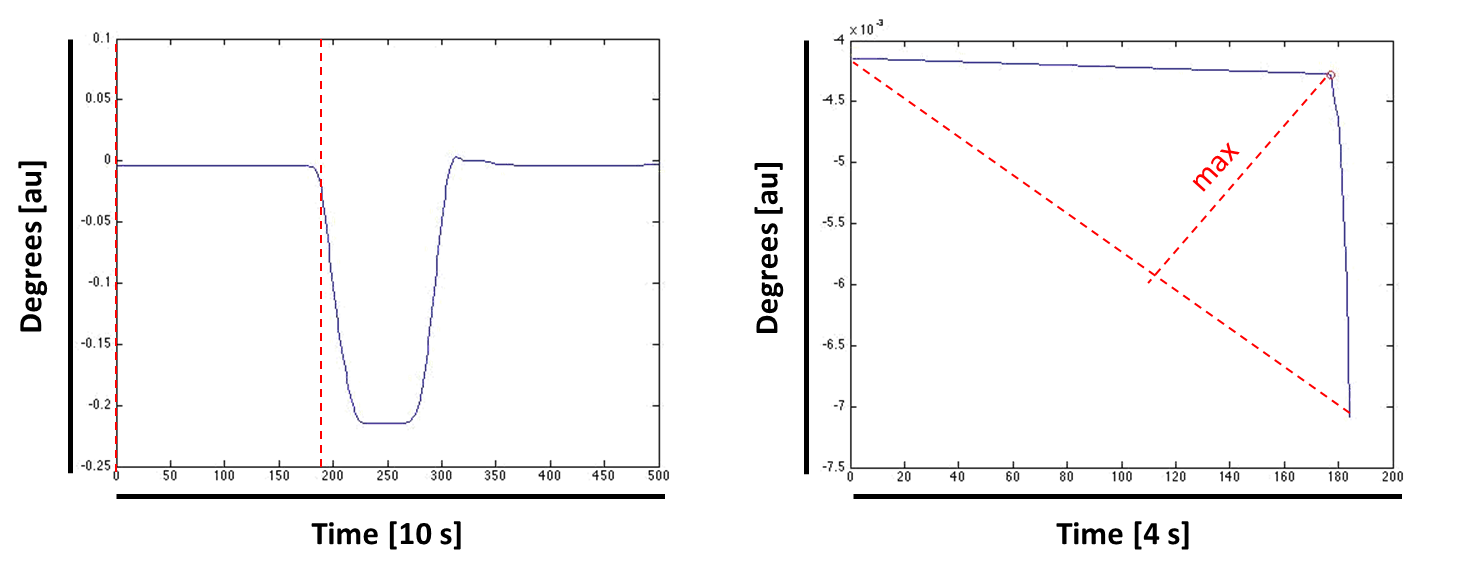
**

**Supplementary Fig.1** Left panel: non-ecological trial showing the whole excursion of the steering wheel from the presentation of the turning sign (time, 0 s) to the end of the trial (time, 10 s). Dashed red line limit the analysis time window used to identify the steering onset. Right panel: depiction of the algorithm used to detect the steering onset as descried in Material and Methods.


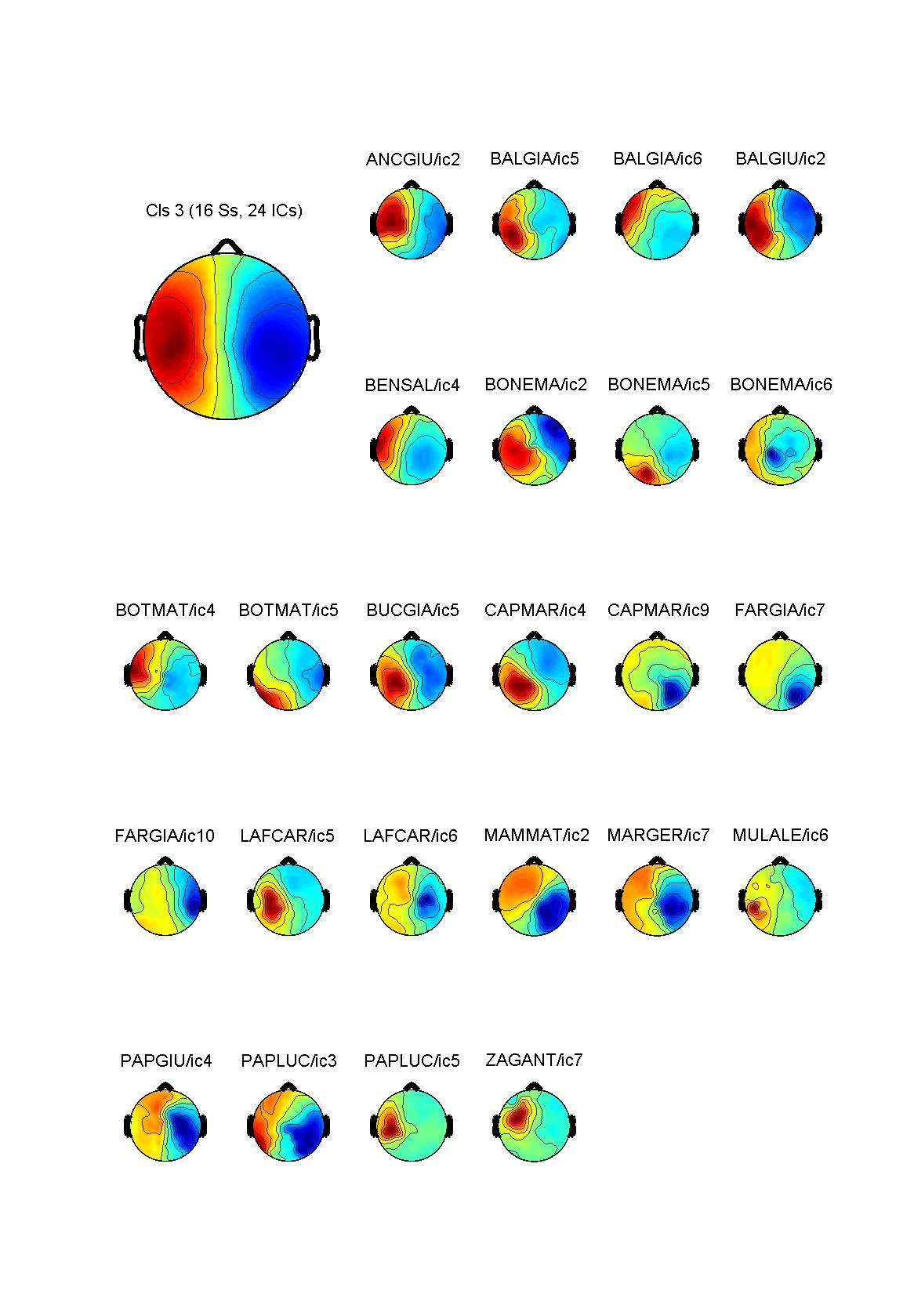


**Supplementary Fig.2.** Average and individual topographies for cluster 3. The average scalp topography of cluster 3 is shown in the upper left part of the picture. Individual scalp topographies are shown with the participant ID (S) and the related IC belonging to the cluster.


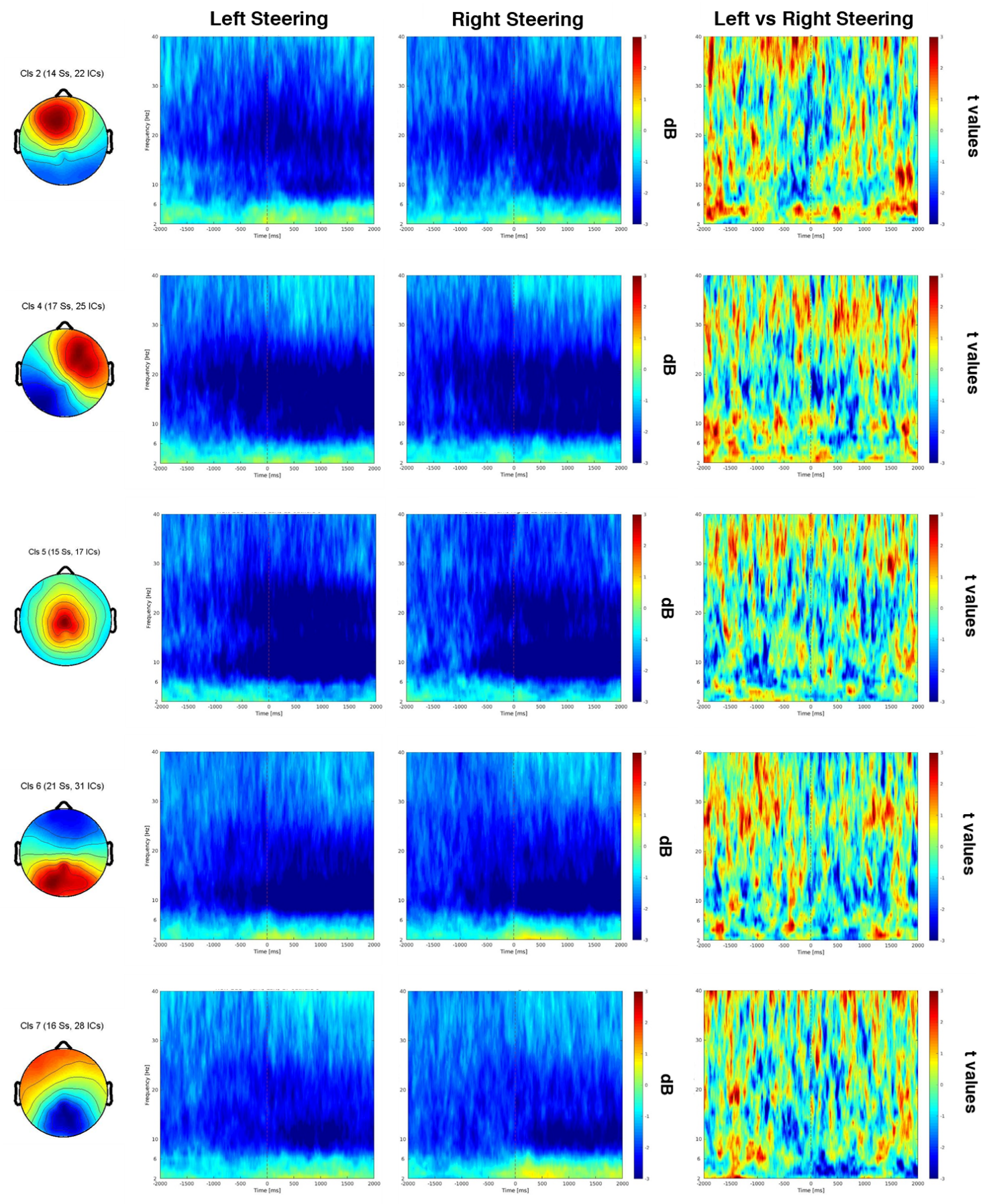


**Supplementary Fig.3** Scalp topography and EEG ERSP of the non-significant cluster resulting from the non-ecological task.
Each row illustrates the EEG ERSP of each cluster for left (left column) and right (middle) steering as well as the statistical comparison of the two conditions (right). The topographies in the left part of the picture show the average scalp maps related to the cluster centroid with the corresponding number of subjects (Ss) and independent component (ICs). Colorbars indicate in blue (red) the desynchronization (synchronization) of the EEG activity (left and middle panels) with respect to the baseline, as well as the statistical differences corresponding to the increase (decrease) of such activity during the left (right) steering (right panels)


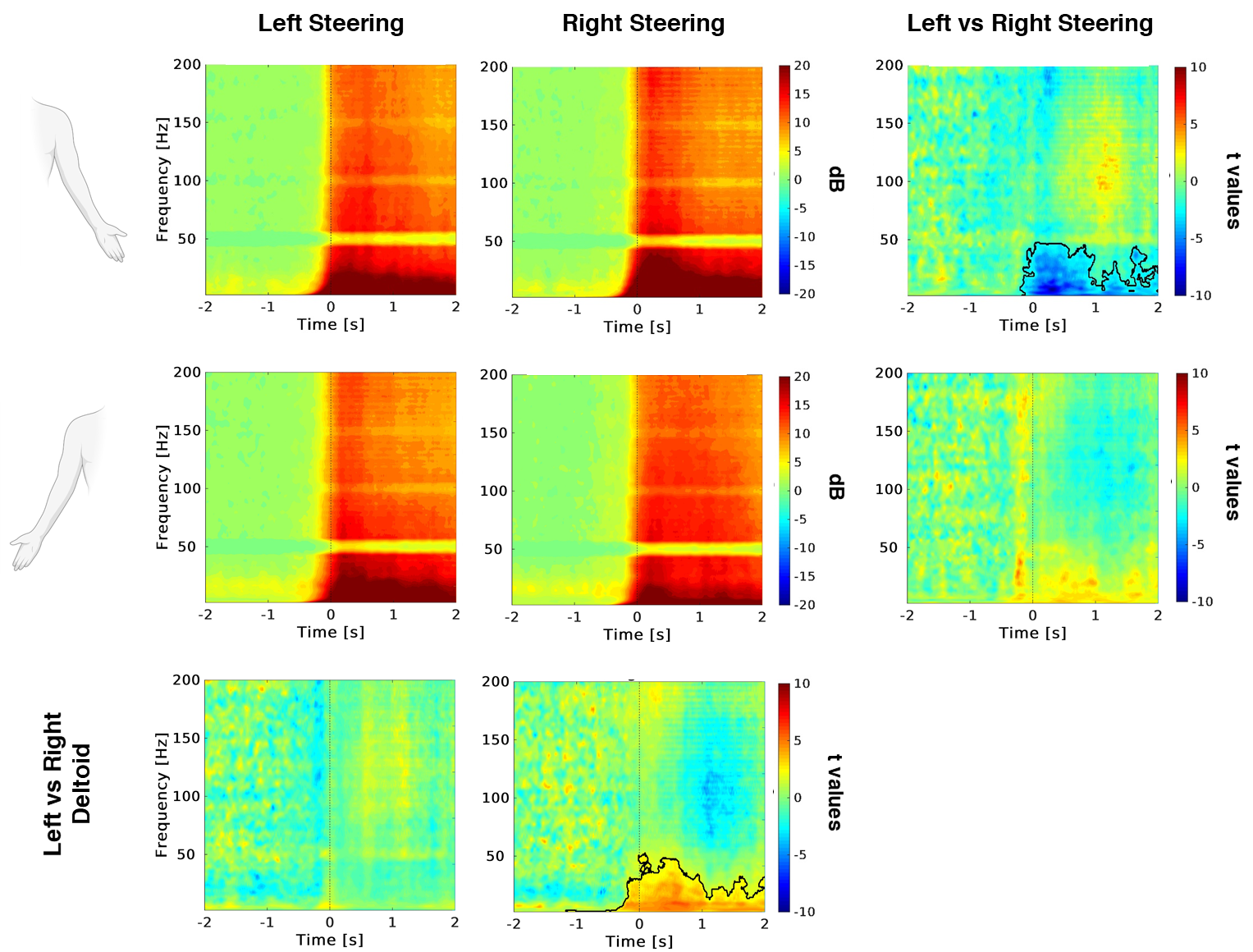


**Supplementary Fig.4** EMG ERSP computed for the forearm extensors during the non-ecological steering task. First (second) row (from the top) illustrates the EMG ERSP for the left (right) forearm extensor during left (left panels) and right (middle panels) steering as well as the statistical comparison of the two conditions (right panels). Third row illustrates the statistical comparisons of the EMG ERSP between left and right forearm extensor during left (left panel) and right (right panel) steering. Colorbars indicate in red (blue) the synchronization (relaxation) of the EMG activity (inner panels) with respect to the baseline, as well as the increase (decrease) of such activity during the left (right) steering for the right (left) deltoid, as well as for the left (right) deltoid during right (left) steering (outer panels)

**EEG data analysis and results of the ecological steering task**

EEG data during the ecological task were collected and pre-processed following the procedure described in the main text. We performed the independent component analysis (ICA) on this dataset to investigate whether the identified EEG IC reacting to non-ecological steering was also present in the ecological steering task. On average, we identified 5.6 (± 2.2) independent components (ICs) per subject for a total sum of 135 EEG ICs. The K-means algorithm performed a cluster analysis to group components into 6 clusters according to their scalp topographies. For each of these ICs and steering conditions (i.e., left and right), we computed the ERSP and statistically compared the corresponding time-frequency panels (i.e., left vs. right steering), following the procedure described in the main text. Results are illustrated in Supplementary Fig.4, showing no significant variation of EEG activity anticipating steering action for any identified cluster.


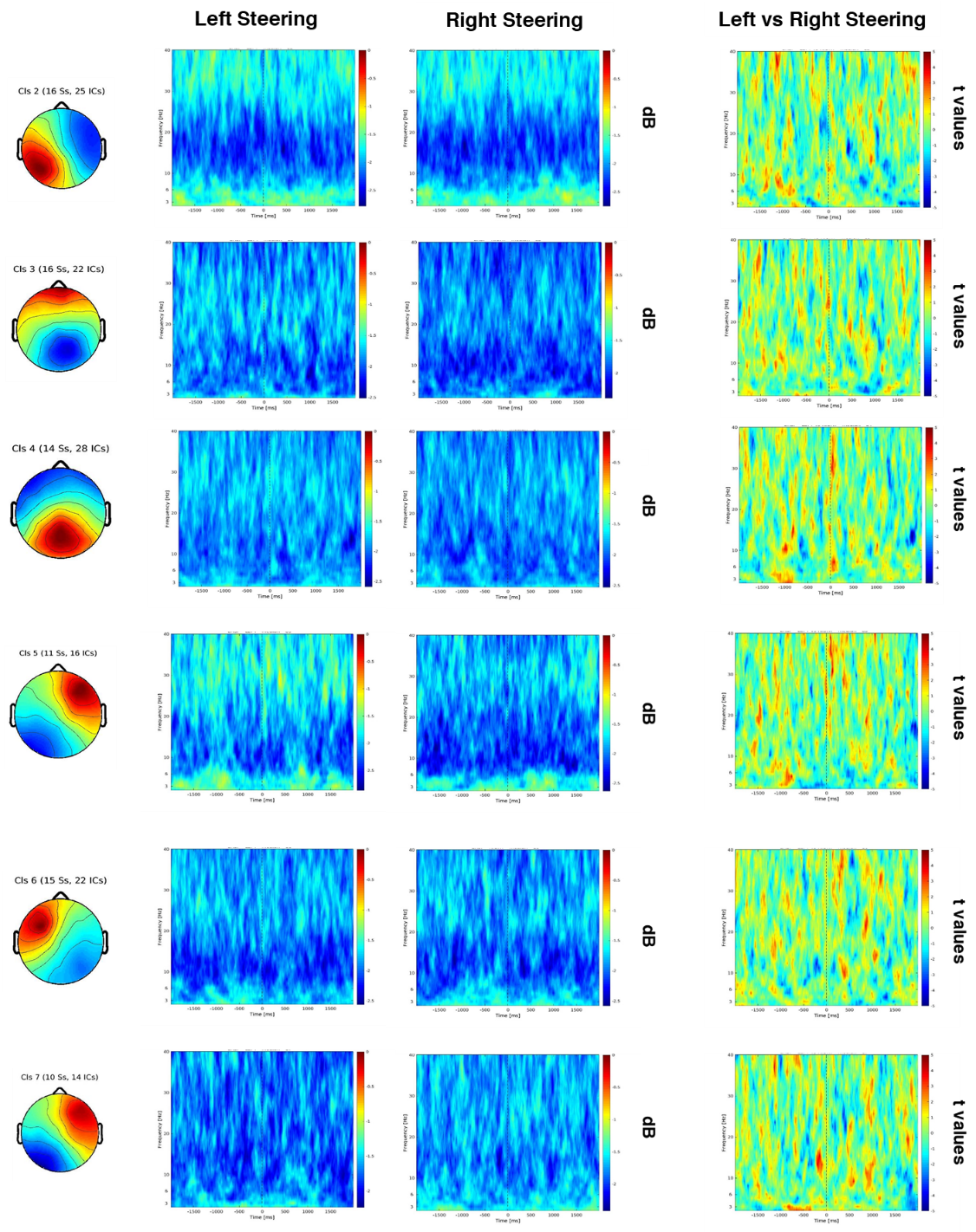


**Supplementary Fig.5** Scalp topography and EEG IC ERSP of the cluster resulting from the ecological task. Each row illustrates the EEG IC ERSP of each cluster for left (left column) and right (middle) steering as well as the statistical comparison of the two conditions (right). The topography in the left part of the picture shows the average scalp map related to the cluster centroid with the corresponding number of subjects (Ss) and independent component (ICs). Colourbars indicate in blue (red) the desynchronization (synchronization) of the EEG activity (left and middle panels) with respect to the baseline, as well as the statistical differences corresponding to the increase (decrease) of such activity during the left (right) steering (right panels).
